# Causal inference of regulator-target pairs by gene mapping of expression phenotypes

**DOI:** 10.1186/1471-2164-7-125

**Published:** 2006-05-24

**Authors:** David C Kulp, Manjunatha Jagalur

**Affiliations:** 1 Bioinformatics Research Lab, Department of Computer Science, University of Massachusetts, Amherst, MA, USA

## Abstract

**Background:**

Correlations between polymorphic markers and observed phenotypes provide the basis for mapping traits in quantitative genetics. When the phenotype is gene expression, then loci involved in regulatory control can theoretically be implicated. Recent efforts to construct gene regulatory networks from genotype and gene expression data have shown that biologically relevant networks can be achieved from an integrative approach. In this paper, we consider the problem of identifying individual pairs of genes in a direct or indirect, causal, *trans*-acting relationship.

**Results:**

Inspired by epistatic models of multi-locus quantitative trait (QTL) mapping, we propose a unified model of expression and genotype to identify quantitative trait genes (QTG) by extending the conventional linear model to include both genotype and expression of regulator genes and their interactions. The model provides mapping of specific genes in contrast to standard linkage approaches that implicate large QTL intervals typically containing tens of genes. In simulations, we found that the method can often detect weak *trans*-acting regulators amid the background noise of thousands of traits and is robust to transcription models containing multiple regulator genes. We reanalyze several pleiotropic loci derived from a large set of yeast matings and identify a likely alternative regulator not previously published. However, we also found that many regulators can not be so easily mapped due to the presence of *cis*-acting QTLs on the regulators, which induce close linkage among small neighborhoods of genes. QTG mapped regulator-target pairs linked to ARN1 were combined to form a regulatory module, which we observed to be highly enriched in iron homeostasis related genes and contained several causally directed links that had not been identified in other automatic reconstructions of that regulatory module. Finally, we also confirm the surprising, previously published results that regulators controlling gene expression are not enriched for transcription factors, but we do show that our more precise mapping model reveals functional enrichment for several other biological processes related to the regulation of the cell.

**Conclusion:**

By incorporating interacting expression and genotype, our QTG mapping method can identify specific regulator genes in contrast to standard QTL interval mapping. We have shown that the method can recover biologically significant regulator-target pairs and the approach leads to a general framework for inducing a regulatory module network topology of directed and undirected edges that can be used to identify leads in pathway analysis.

## Background

Recently several data sets of both whole genome genotype and expression data have been published [1-4]. In a major departure from conventional genetic trait analysis, now 10^5 ^to 10^6 ^phenotypic traits are represented by distinct gene expression measurements. Theoretically, chromosomal regions can be linked to the expression of each of the many measured genes. Thus, this data provides the basis for determining the role of genetic variation in differential gene expression and the identification of polymorphic genes that regulate, directly or indirectly, transcriptional control.

### Genetics of gene expression

The three most significant studies to date include whole genome expression and genotyping for a collection of 113 segregants from a mating of two isogenic yeast strains [5], a collection of 111 selfed progeny from a mating of two inbred mice strains [2], and 32 recombinant inbred mice strains [6]. The yeast regulatory system is simpler, recombination rates are higher, and the number of available samples is greater than in the recombinant inbred mouse dataset currently available; therefore, we focus on the work of Brem and colleagues exclusively. With sufficient sampling, the principles of our work should be applicable to other model systems.

Quantitative trait loci (QTL) are regions on a genome in which the genetic variation is significantly correlated with a phenotypic trait, here the expression of a gene. *Interval mapping *[7] models assume an additive relationship of genotype to phenotype according to a mixture model of the form:

*P*(*T*_*i*_|*Q*_*j*_) = N
 MathType@MTEF@5@5@+=feaafiart1ev1aaatCvAUfKttLearuWrP9MDH5MBPbIqV92AaeXatLxBI9gBamrtHrhAL1wy0L2yHvtyaeHbnfgDOvwBHrxAJfwnaebbnrfifHhDYfgasaacH8akY=wiFfYdH8Gipec8Eeeu0xXdbba9frFj0=OqFfea0dXdd9vqai=hGuQ8kuc9pgc9s8qqaq=dirpe0xb9q8qiLsFr0=vr0=vr0dc8meaabaqaciaacaGaaeqabaWaaeGaeaaakeaaimaacqWFneVtaaa@383B@(*β*_0 _+ *β*_1_*Q*_*j*_, *σ*)

where *T*_*i *_is the expression of the *i*^*th *^transcript and *Q*_*j *_is a numeric genotype value corresponding to the *j*^*th *^site in the genome. (Genotypes usually take on three values, -1, 0, and 1 for biallelic sites in diploids. Genotypes for haploid or back-crossed individuals are only two-valued, i.e. 0 and 1.) For an observed *T*_*i*_, all possible *j *are considered and the *β *terms fit by regression. When measured markers are sparse, *Q*_*j *_is usually sampled in regular intervals between the markers, in which case, *Q*_*j *_is estimated according to a maximum likelihood approach. Consecutive regions along a chromosome with log odds (LODscore=log⁡10P(Ti|Qj,β0,β1,σ)P(Ti|Qj,β0,β1=0,σ)
 MathType@MTEF@5@5@+=feaafiart1ev1aaatCvAUfKttLearuWrP9MDH5MBPbIqV92AaeXatLxBI9gBaebbnrfifHhDYfgasaacH8akY=wiFfYdH8Gipec8Eeeu0xXdbba9frFj0=OqFfea0dXdd9vqai=hGuQ8kuc9pgc9s8qqaq=dirpe0xb9q8qiLsFr0=vr0=vr0dc8meaabaqaciaacaGaaeqabaqabeGadaaakeaaieaacqWFmbatcqWFpbWtcqWFebarcqWFGaaicqWFZbWCcqWFJbWycqWFVbWBcqWFYbGCcqWFLbqzcqGH9aqpcyGGSbaBcqGGVbWBcqGGNbWzdaWgaaWcbaGaeGymaeJaeGimaadabeaakmaalaaabaGaemiuaaLaeiikaGIaemivaq1aaSbaaSqaaiabdMgaPbqabaGccqGG8baFcqWGrbqudaWgaaWcbaGaemOAaOgabeaakiabcYcaSGGaciab+j7aInaaBaaaleaacqaIWaamaeqaaOGaeiilaWIae4NSdi2aaSbaaSqaaiabigdaXaqabaGccqGGSaalcqGFdpWCcqGGPaqkaeaacqWGqbaucqGGOaakcqWGubavdaWgaaWcbaGaemyAaKgabeaakiabcYha8jabdgfarnaaBaaaleaacqWGQbGAaeqaaOGaeiilaWIae4NSdi2aaSbaaSqaaiabicdaWaqabaGccqGGSaalcqGFYoGydaWgaaWcbaGaeGymaedabeaakiabg2da9iabicdaWiabcYcaSiab+n8aZjabcMcaPaaaaaa@6850@) greater than some threshold are identified as candidate QTL intervals, within which are genes believed responsible for the phenotypic variation. In our case, each QTL interval putatively contains a regulator gene. The identification of the specific regulator gene is referred to as *fine mapping*.

In Brem [4], expression levels were measured for 5727 ORFs and genotype data obtained for 2957 markers regularly spaced across the yeast chromosomes. They found that a large fraction of differential gene expression was due to genetic variation and Yvert, et al [8] showed that, perhaps surprisingly, the genes in the QTL intervals were not enriched for transcription factors or any particular gene function. This observation could possibly be explained by the large size of the QTL intervals, typically containing ten or more genes, however we will show that our more precise models still do not implicate transcription factors as dominant upstream regulators. Thus, one must conclude that regulatory influence is a complex process such that "upstream regulator" is interpreted in the broadest of contexts.

Polymorphisms affecting gene expression are conventionally divided into *cis*- and *trans*-acting effects, i.e. polymorphisms that are proximal to the gene, such as in the promoter or 3' end, and those in another gene (generally speaking on a different chromosome). Detecting *cis*-acting QTLs is straightforward using interval mapping and such QTLs tend to be highly significant [2]. On the other hand, *trans*-acting variation accounts for most differential gene expression, but the variation is due to a large number of weak actors [5]. This is consistent with a model of gene regulation in which multiple factors contribute in macromolecular complexes and in many different stages of cell regulation such as signaling, transport, and so on.

### Inferring regulatory networks from correlated gene expression

Independent of the data sets described so far, large collections of gene expression over time course [9] or varying environmental conditions [10,11] have been studied to reveal dependent variation among genes and thereby deduce regulatory relationships. A dominant model used in such analyses was first proposed by Friedman, et al [12] in which each gene is a random variable with conditional distribution dependent on a small number of parent variables according to the Bayesian network (BN) formalism.

In such a model, nodes in a graph are random variables representing gene expression and edges connect nodes in a directed acyclic graph (DAG).

In the BN modeling method, the key design factors are (1) the estimation of the conditional probability term *P*(*T*_*i*_|*Pa*(*T*_*i*_)) – abstractly a score function, where *Pa*(*T*_*i*_) are the parents of gene *T*_*i *_– and (2) an efficient means of discovering the set *Pa*(*T*_*i*_). Both parametric continuous and non-parametric discrete score functions have been considered. The discrete case is common in the literature; relative gene expression is discretized usually into categorical increased, decreased, and unchanged values and conditional probability tables (CPT) are constructed from tallied observations of the values among parents and child. A CPT model can theoretically capture complex relationships among the parents, but this power is usually limited by the binning of expression values into a few values in order to achieve adequate conditional density estimates.

Continuous models are attractive because parameters are estimated from the totality of the data, but computational efficiency concerns have conventionally limited the class of models considered to simple linear Gaussian models with a small number of parameters of the form

P(Ti|Pa(Ti))=N(β0+∑j∈Pa(Ti)βjTj,σ).
 MathType@MTEF@5@5@+=feaafiart1ev1aaatCvAUfKttLearuWrP9MDH5MBPbIqV92AaeXatLxBI9gBamrtHrhAL1wy0L2yHvtyaeHbnfgDOvwBHrxAJfwnaebbnrfifHhDYfgasaacH8akY=wiFfYdH8Gipec8Eeeu0xXdbba9frFj0=OqFfea0dXdd9vqai=hGuQ8kuc9pgc9s8qqaq=dirpe0xb9q8qiLsFr0=vr0=vr0dc8meaabaqaciaacaGaaeqabaWaaeGaeaaakeaacqWGqbaucqGGOaakcqWGubavdaWgaaWcbaGaemyAaKgabeaakiabcYha8jabdcfaqjabdggaHjabcIcaOiabdsfaunaaBaaaleaacqWGPbqAaeqaaOGaeiykaKIaeiykaKIaeyypa0dcdaGae8xdX7KaeiikaGccciGae4NSdi2aaSbaaSqaaiabicdaWaqabaGccqGHRaWkdaaeqbqaaiab+j7aInaaBaaaleaacqWGQbGAaeqaaOGaemivaq1aaSbaaSqaaiabdQgaQbqabaGccqGGSaalcqGFdpWCcqGGPaqkaSqaaiabdQgaQjabgIGiolabdcfaqjabdggaHjabcIcaOiabdsfaunaaBaaameaacqWGPbqAaeqaaSGaeiykaKcabeqdcqGHris5aOGaeiOla4caaa@6210@

Each parent adds an independent contribution to *T*_*i*_, eliminating the potential interacting effects among parents. But interacting effects can be important under limited circumstances and we will suggest below a relaxed Gaussian model that is computationally acceptable.

The major drawback of the BN approach for analyzing gene expression data alone is that the dependencies inferred among variables do not necessarily imply causality. Indeed, while it is possible to determine causality in some cases [13], in general, for any BN solution there is an equivalence class represented by a partially directed acyclic graph (PDAG) of alternative solutions with different edge directions that have the same joint probability.

Pe'er, et al [14] describe how to infer causality under certain gene expression perturbation experiments. (In some sense, our genotype/expression data sets represent the ideal randomized perturbation experimental design, where allelic expression is perturbed by random recombination.) Inother work, transcription factor binding sites (either predicted or experimentally determined) have been added to infer direction of regulation [15,16]. A limitation of these approaches is that strong priors over allowable structures are implicitly or explicitly applied, which limits the relationships that may be discovered. For example, Segal, et al [16] limit their search to downstream targets of a small set of known transcription factors. However, gene expression regulation is much more complex, involving other direct and indirect factors such as post-translational modification or protein signaling cascades.

(Note that BNs also lack cycles that exist in real biological networks. This can be overcome using Dynamic Bayesian Networks [17] when time course data is available, which is not the case here.)

### Previous work

Zhu, et al [18] describes a method to build comprehensive BN reconstructions of regulatory networks based on genotype/expression data sets. Their work is motivated, as we are, by the recognition that correlation between genotype and expression *does *imply causality. Genotype assignments represent random shuffling during meiosis, so correlations observed must be the effect of causative polymorphisms. Their approach is to weight the BNs with priors according to rules regarding the chromosomal positions of genes and the differences in QTLs between pairs of genes. A key idea in this work is that related genes will share multiple QTLs. More recently, the same group has described a protocol for inferring the causal nature between two traits – in particular, their method can be applied to the expression levels of a pair of genes [19]. In their approach, pairs of traits that share multiple QTLs are tested for causal orientation based on independence conditioned on the genotypes of the shared QTLs.

Li, et al [20] also addressed this problem by filtering the set of candidate parent genes of a target gene to only those genes with coding SNPs located within QTL intervals with stringent LOD scores. With a much smaller set of possible model configurations it was possible to exhaustively search all BN configurations using a score function based on gene expression alone.

Lastly, in a similar strategy, Bing and Hoeschele [21] recommend an analysis protocol for genotype/expression data in which individual genes within a QTL interval are considered as parents according to their expression correlation to the target gene.

In all of the above cited works, regulatory relationships are derived roughly according to a two-step process in which standard QTL interval mapping is first applied – which serves to filter the set of parent genes – followed by a selection of regulator-target pairs according to gene expression correlation or conditional independence tests. It is conceivable that the interaction between polymorphisms in and expression of a regulator may have a significant effect, not observed by either factor alone. We propose such a simple, unified model for the scoring of candidate regulator-target pairs that considers all scenarios of *cis- *and *trans- *effects, allowing for interaction among gene expression and genotype. (Note that we do not model the interacting effects of multiple QTLs (epistasis) [22]. While it has been estimated that at least 14% of genes are controlled by epistatic effects between two simultaneous linkages, the ability to detect such pairs in small sample sets is very limited [23].)

## Results

### Function enrichment

As with networks derived from gene expression alone, connectivity between genes does not necessarily imply physical interactions. Yvert, et al previously observed that genes within QTLs of gene expression traits were not enriched for transcription factors or any other function [8]. Nevertheless, we wondered whether this lack of functional enrichment was due to the imprecise mapping of intervals that contain usually tens of candidate genes. We hypothesized that our QTG mapping method, which identifies specific candidate genes, might show enrichment for transcription factors or other functional categories.

To test this hypothesis, we analyzed the yeast set consisting of 6164 gene expression measurements and 2957 genotype markers across 113 matings between two distinct isogenic strains [5]. We computed the pairwise dependency among all pairs of genes according to the full and reduced model scores, selecting those pairs with a *p *< 0.00001 based on exhaustive permutation tests (required for each pair for the full model and expression reduced-model). This resulted in 22,923 predicted interacting pairs yielding a modest false discovery rate of 1.7%. Finally, to avoid linkage disequilibrium effects, putative *cis*-*trans*-acting regulators (using a conventional 0.05 p-value cutoff) were excluded and regulator-target pairs residing on the same chromosome were removed. This filtering likely removed some true pairs, but we chose to favor conservative selection in order to detect any group-wide trends that would be obscured by noise from false positives. Our final set consisted of 4268 pairs.

We then considered the significance of each Gene Ontology (GO [24]) category in the "biological process" and "molecular function" ontologies with respect to the known GO assignments to the candidate regulators using the standard hypergeometric distribution test. Unlike previous reports, we found some highly significant classes shown in table 1. However, we did not find enrichment among transcription factors or related activity, in agreement with Yvert, et al [8]. It is interesting that there is enrichment in many different regulatory and control related activities, including cell cycle regulation, metabolism, and kinase activity, but most enrichment is for functions and processes related to protein translation. Ribosomal proteins and related genes are well known to be highly co-expressed, but this analysis supports the stronger claim that these genes are auto-regulated to a high degree [25].

**Table 1:** Functional enrichment of regulators in GO. The set of GO terms from the "molecular function" (F) and "biological process" (P) categories showing significant enrichment among the candidate QTG. Total number of genes in yeast genome is 6164. Total number of regulators in filtered set is 823.

# in genome	# of regulators	P-value	GO Type	GO ID	GO Term
216	50	10^-30^	F	GO:0003735	structural constituent of ribosome
264	57	10^-29^	P	GO:0006412	protein biosynthesis
60	19	10^-22^	P	G0:0006364	rRNA processing
62	19	10^-21^	P	G0:0006365	35S primary transcript processing
46	14	10^-16^	P	G0:0030490	processing of 20S pre-rRNA
39	12	10^-14^	P	G0:0000027	ribosomal large subunit assembly and maintenance
91	19	10^-12^	P	GO:0006468	protein amino acid phosphorylation
145	27	10^-12^	F	G0:0003723	RNA binding
8	5	10^-11^	P	G0:0006109	regulation of carbohydrate metabolism
25	8	10^-11^	P	G0:0000074	regulation of cell cycle
14	6	10^-10^	P	G0:0000183	chromatin silencing at ribosomal DNA
33	9	10^-10^	F	G0:0003899	DNA-directed RNA polymerase activity
53	12	10^-10^	F	G0:0004672	Protein kinase activity

Even though no functional enrichment in transcription factors was found, we still examined the predicted targets of transcription factors for evidence of physical interaction. Considering all the predicted targets of each transcription factor that met the selection criteria above, we searched 500nts upstream of the target for matches to known binding site motifs (TRANSFAC [26]). We found no significant enrichment for targets containing known binding regardless of sequence similarity thresholds. For example, only 35 of 719 putative targets contained matches to known binding sites. And of those, only 8 were known targets of their respective transcription factor regulators.

In a final attempt to recover a bias for transcription factors, we hypothesized that QTGs associated with multiple target genes would be enriched for transcription factors. We extracted those regulators from our set of 4268 pairs that had ten or more target genes. The set included well known transcription factors FKH1, FKH2, MSN1, KSP1, and ZAP1, but there was no significant enrichment in the total set for transcriptional regulators. All these observations further confirm that regulatory behavior captured in genotype/expression networks is not likely to be physical interactions, but more complex, indirect relationships as suggested by the functional enrichment found above.

### Robustness

Next we wondered how well a causal relationship could be inferred when the regulator was part of a multi-factor regulon. Using the yeast data set of *n *= 6164 genes, we simulated an *n *+ 1 target gene according to an additive model of *k *= 2 ... 5 regulators, with only one regulator having genotypic effect. Specifically, we simulated

*T*_*n*+1 _= *β*_1_*T*_1 _+ ... + *β*_*k*_*T*_*k *_+ *β*_*k'*_*T*_*k*_*Q*_*k *_+ *ε*

where *β*_*k' *_was set at random values such that the genotypic effect between the two alleles, (*μ*_*a *_- *μ*_*b*_)/*σ*, was uniformly selected between 0.5 and 3.0. The other *β*'s were selected from N
 MathType@MTEF@5@5@+=feaafiart1ev1aaatCvAUfKttLearuWrP9MDH5MBPbIqV92AaeXatLxBI9gBamrtHrhAL1wy0L2yHvtyaeHbnfgDOvwBHrxAJfwnaebbnrfifHhDYfgasaacH8akY=wiFfYdH8Gipec8Eeeu0xXdbba9frFj0=OqFfea0dXdd9vqai=hGuQ8kuc9pgc9s8qqaq=dirpe0xb9q8qiLsFr0=vr0=vr0dc8meaabaqaciaacaGaaeqabaWaaeGaeaaakeaaimaacqWFneVtaaa@383B@(0,1). Using the QTG *trans *model we attempted to recover the *causal *regulator of the simulated target among the background of the other *n *genes. By modifying the full model threshold for equation 2 we obtained different tradeoffs between recall and precision. We compared this approach to the standard QTL mapping approach and to a more liberal, but realistic, test in which we defined a true positive as correctly identifying the *interval *containing the regulator (false positives were intervals not containing the true regulator). We found that our QTG model was successful in identifying the correct regulating gene, even for larger values of *n *(figure 4). Not surprisingly, conventional QTL mapping alone, being a function of only the flanking markers, failed to accurately predict the precise regulating gene, but the QTL interval was typically identified with reasonable success in our simulation.

### Prediction of novel regulators

We next considered six candidate QTL intervals analyzed in [4] representing highly pleiotropic loci. The intervals were each predicted as containing a regulator gene associated with a large number of target genes, although the precise gene was unknown. In the paper, a putative gene within each interval was predicted manually by the authors according to published gene function annotations of regulator and target genes. For the most part, QTG analysis was disappointing in these cases; as it turned out, loci 1 through 5 were coincident with cis-acting QTLs. As a result, the gene expression of most of the putative regulators are highly linked and the manually predicted genes are no better fit than the neighboring genes.

However, we predicted a likely alternative regulator for the second of the six loci. The region on chromosome III represented a common QTL for 21 genes identified by Brem, who predicted that LEU2 was the putative regulator based on its similar function to these 21 target genes. But we identified ILV6, about 13 kb from LEU2, as the more likely candidate. ILV6 is the best fit for the full and reduced models for 12 of the 21 genes with no other candidate gene showing significant fit for more than a few targets. Scanning the genome, we also found an additional five target genes not previously identified (table 2). This set of 17 putative targets of ILV6 are significantly enriched for genes associated with branched chain family amino acid biosynthesis (p-value 1.8 × 10^-8^) and related amino acid metabolism GO terms. Moreover, ILV6 has been shown through direct assays to be part of the super-pathway for leucine, isoleucine, and valine as the regulatory sub-unit of acetolactate synthase [27]. Thus, ILV6 and its targets are functionally related and it is highly plausible that modulation of ILV6 directly affects the abundance of these other genes.

**Table 2:** ILV6 targets. The putative targets of a novel regulator gene, ILV6, in the "group 2" loci from [4]. The list includes five additional targets not previously identified.

Novel	Target Gene	QTG Score	QTL Score	Description
Y	YLR040C	49	35	hypothetical protein
Y	STE3	43	36	Receptor for a factor receptor, transcribed in alpha cells and required for mating by alpha cells, couples to MAP kinase cascade to mediate pheromone response; ligand bound receptors are endocytosed and recycled to the plasma membrane; GPCR,
N	LEU4	29	7	Alpha-isopropylmalate synthase (2-isopropylmalate synthase); the main isozyme responsible for the first step in the leucine biosynthesis pathway
N	ILV3	24	7	Dihydroxyacid dehydratase, catalyzes third step in the common pathway leading to biosynthesis of branched-chain amino acids
N	ILV2	22	7	Acetolactate synthase, catalyses the first common step in isoleucine and valine biosynthesis and is the target of several classes of inhibitors, localizes to the mitochondria; expression of the gene is under general amino acid control
N	SNZ1	20	6	Protein involved in vitamin B6 biosynthesis; member of a stationary phase-induced gene family; coregulated with SNO1; interacts with Snolp and with Yhrl98p, perhaps as a multiprotein complex containing other Snz and Sno proteins
N	ALD5	20	7	Mitochondrial aldehyde dehydrogenase, involved in regulation or biosynthesis of electron transport chain components and acetate formation; activated by K+; utilizes NADP+ as the preferred coenzyme; constitutively expressed
N	BAT1	19	9	Mitochondrial branched-chain amino acid aminotransferase, homolog of murine ECA39; highly expressed during logarithmic phase and repressed during stationary phase
N	OAC1	19	10	Mitochondrial inner membrane transporter, transports oxaloacetate, sulfate, and thiosulfate; member of the mitochondrial carrier family
N	YLR089C	18	4	Putative alanine transaminase (glutamic pyruvic transaminase)
N	DIC1	18	7	Mitochondrial dicarboxylate carrier, integral membrane protein, catalyzes a dicarboxylate-phosphate exchange across the inner mitochondrial membrane, transports cytoplasmic dicarboxylates into the mitochondrial matrix
N	BAP2	17	9	High-affinity leucine permease, functions as a branched-chain amino acid permease involved in the uptake of leucine, isoleucine and valine; contains 12 predicted transmembrane domains
N	YOR225W	16	10	
Y	CTF13	15	4	Subunit of the CBF3 complex, which binds to the CDE III element of centromeres, bending the DNA upon binding, and may be involved in sister chromatid cohesion during mitosis
Y	SN01	15	4	Protein of unconfirmed function, involved in pyridoxine metabolism; expression is induced during stationary phase; forms a putative glutamine amidotransferase complex with Snzlp with Snolp serving as the glutaminase
N	ISU2	14	5	Conserved protein of the mitochondrial matrix, required for synthesis of mitochondrial and cytosolic iron-sulfur proteins, performs a scaffolding function in mitochondria during Fe/S cluster assembly; isu1 isu2 double mutant is inviable
Y	PMI40	9	4	Mannose-6-phosphate isomerase, catalyzes the interconversion of fructose-6-P and mannose-6-P; required for early steps in protein mannosylation

### Prediction of novel structure

Finally, we constructed a putative network from the seed gene, ARN1, as a demonstration of the characteristics of a regulatory module that might be derived from our models. Regulatory modules of the iron homeostasis pathway have been previously constructed from the gene expression data of Hughes, et al [11,14,28,29].

For the purposes of this work, we consider networks constructed simply according to the pairwise relationships; that is, the network is not a BN, but its edges are derived from Markov pairs according to our derived score functions. In order to concretely demonstrate the advantage of incorporating the variation data and compare with a structure that might be derived from an approach such as by Pe'er et al, we also constructed a BN from the Markov Blanket of ARN1 [30]. The BN structure was constructed using the K2 algorithm on discretized gene expression values [31].

Most genes in our module reconstruction in figure 5 are iron homeostasis and many that were found were common to previous reconstructions based on a different expression data set. Both the reductive mechanism directly associated with ARN1 (ARN2, SIT1, FTR1, SMF3, FITS) and the non-reductive transport (FRE6) mechanism were implicated in the network. Interestingly, two key genes in this pathway, FTR1 and FET3, although not directly linked to ARN1, are both involved in iron uptake and were found to have significant *bi*-directional causality, implying an auto-regulatory mechanism.

## Methods

**Figure 1:**
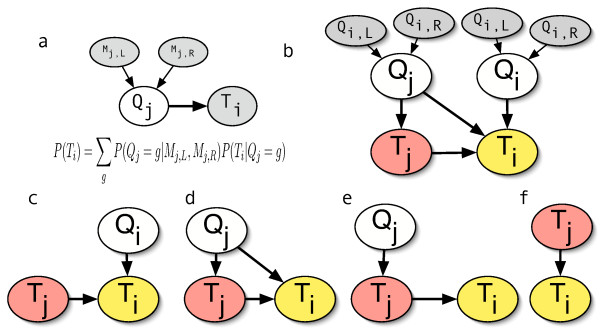
**Graphical model representation**. (a) The representation of conventional interval mapping as a graphical model. For an observed *i*, all candidate genotype sites *j *are considered. (b) The QTG model of a single regulator-target pair of genes (regulator is gene *j *and target is gene *i*). Subgraphs of (b) represent (c) *cis*-, (d) *trans*-, (e) *cis-trans*- cases, and (f) no genotypic effect, corresponding to the conventional BN. Colored and shaded nodes are observed.

We represent the genotypes and the expression measures as numeric random variables in a graphical model. In the general case of QTL interval mapping using sparse marker data, the genotype at a site of interest is an unknown random variable, *Q*_*j*_, dependent on the observed genotypes of the nearest upstream and downstream flanking markers, *M*_*j*,*L*_,*M*_*j*,*R*_. The conditional probability of the unobserved genotype is a well-known function of the recombination distances among *Q*_*j*_, and *M*_*j*,*L*_,*M*_*j*,*R *_[32]. Assuming that some observed phenotype (here gene expression, *T*_*i*_, where *i *ranges over the number of genes) is dependent on *Q*_*j*_, then the graphical model is shown in figure 1a. QTL interval mapping is then the maximum likelihood estimate of each *Q*_*j *_and the selection of those *Q*_*j *_where the log likelihood exceeds some threshold.

We are concerned with the class of *trans*-acting regulators in which the expression of the target is dependent on the expression of the regulating gene. We consider three sub-classes of genotypic effect: *cis-, trans-*, and *cis*-*trans*-acting sites. For example, a variation in the promoter region or 3' end of the target gene may have a *cis*-acting effect on the expression level of the target; a variation in the coding region of the regulator may have a *trans*-acting effect, either directly or indirectly, on the expression of a target gene, such as through the modification of a DNA-binding motif in a transcription factor; and variation in or around the regulator gene may have a *cis*-acting effect on the regulator's expression which indirectly affords a *trans*-acting effect on the target, i.e. *cis-trans*. We make no specific assumptions in our model regarding the precise mechanism of the allelic effect even though it is convenient to imagine examples of transcription factor binding. Variation can have direct or indirect effects on transcript abundance through a variety of mechanisms such as protein levels, RNA degradation rates, splicing, and so on.

If we consider only the genotype sites at the locations of the protein-coding genes in a fully annotated genome, then we can conveniently reference both genotypes and genes with a common index, i.e. *Q*_*i *_represents the genotype for the gene *i *with expression *T*_*i*_. Figure 1b naturally follows. We refer to this model as the full **QTG **model for a single *quantitative trait gene *and the process of estimating regulatory genes for a given target as "QTG mapping". The three genotype sub-classes are subgraphs of the full model shown in figure 1c–f.

### Case 1: trans-acting Regulator

**Figure 2:**
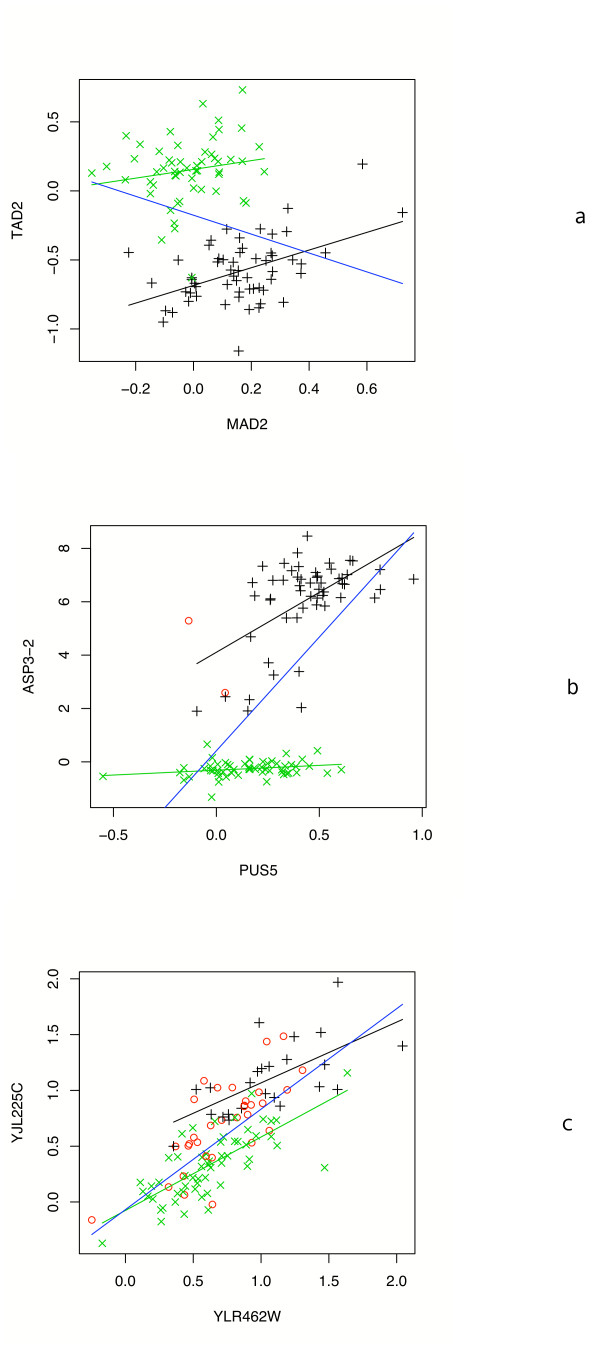
**Interacting effects**. Three examples of the combined and interactive effects of the genotype and expression of a regulator gene on target gene expression in yeast. X and Y axes are expression of regulator and target, respectively. + and × are the two genotypes. Open circles are ambiguous genotypes when flanking markers differ. Regression lines are drawn for expression alone (blue) and by genotype (black and green). For the first example, the regulation and target gene expression appear anti-correlated, but are correlated with respect to genotype. The second example shows the importance of an interacting term to capture the change in the slope. The third example shows significant overlap in the range of target expression for the two alleles, but a clear separation with respect to regulator expression and genotype.

In this paper we address only the *trans*-acting regulator sub-class of figure 1d where the target is dependent on both the genotype and expression of the regulator. It is important to recognize that this is a biologically reasonable scenario with many relevant examples in the data. For example, the scatter plots in figure 2 show the relationships among the expression of a target gene and the expression and genotype of putative regulators. In these cases only the combination, and sometimes interaction, of the regulator's genotype and expression can adequately model the target expression.

Therefore, to consider the possible interactions among genotype and expression, our full model is

*P*(*T*_*i*_|*Q*_*j*_, *T*_*j*_, *θ*) = N
 MathType@MTEF@5@5@+=feaafiart1ev1aaatCvAUfKttLearuWrP9MDH5MBPbIqV92AaeXatLxBI9gBamrtHrhAL1wy0L2yHvtyaeHbnfgDOvwBHrxAJfwnaebbnrfifHhDYfgasaacH8akY=wiFfYdH8Gipec8Eeeu0xXdbba9frFj0=OqFfea0dXdd9vqai=hGuQ8kuc9pgc9s8qqaq=dirpe0xb9q8qiLsFr0=vr0=vr0dc8meaabaqaciaacaGaaeqabaWaaeGaeaaakeaaimaacqWFneVtaaa@383B@(*β*_0 _+ *β*_1_*T*_*j *_+ *β*_2_*Q*_*j *_+ *β*_3_*T*_*j*_*Q*_*j*_, *σ*)     (1)

where *θ *is the *β *and *σ *model parameters.

As with standard interval mapping, Maximum likelihood estimation can be achieved using an expectation maximization (EM) approach in which the genotype, *Q*_*j*_, and the variables, *θ*, are alternatively estimated until convergence. But the advantage of this model over the standard mapping and multi-step approaches previously proposed is that individual loci are automatically mapped in a single step by simultaneously considering all available evidence.

Note that the strength of the genotypic effect is directly related to our ability to infer causality. That is, as the contribution of the *β*_2 _and *β*_3 _terms decreases, our confidence in the causal direction between genes *i *and *j *is reduced. We can be precise about this directionality by comparing our model with the simpler model of no genotypic effect (figure 1f). From equation (1), for each tested gene pair, *i *and *j*, we can determine the strength of a relationship (the *full model score*) as

log⁡10P(Ti|Qj,Tj,θ)P(Ti|Qj,Tj,θ:β1=β2=β3=0)     (2)
 MathType@MTEF@5@5@+=feaafiart1ev1aaatCvAUfKttLearuWrP9MDH5MBPbIqV92AaeXatLxBI9gBaebbnrfifHhDYfgasaacH8akY=wiFfYdH8Gipec8Eeeu0xXdbba9frFj0=OqFfea0dXdd9vqai=hGuQ8kuc9pgc9s8qqaq=dirpe0xb9q8qiLsFr0=vr0=vr0dc8meaabaqaciaacaGaaeqabaqabeGadaaakeaacyGGSbaBcqGGVbWBcqGGNbWzdaWgaaWcbaGaeGymaeJaeGimaadabeaakmaalaaabaGaemiuaaLaeiikaGIaemivaq1aaSbaaSqaaiabdMgaPbqabaGccqGG8baFcqWGrbqudaWgaaWcbaGaemOAaOgabeaakiabcYcaSiabdsfaunaaBaaaleaacqWGQbGAaeqaaOGaeiilaWccciGae8hUdeNaeiykaKcabaGaemiuaaLaeiikaGIaemivaq1aaSbaaSqaaiabdMgaPbqabaGccqGG8baFcqWGrbqudaWgaaWcbaGaemOAaOgabeaakiabcYcaSiabdsfaunaaBaaaleaacqWGQbGAaeqaaOGaeiilaWIae8hUdeNaeiOoaOJae8NSdi2aaSbaaSqaaiabigdaXaqabaGccqGH9aqpcqWFYoGydaWgaaWcbaGaeGOmaidabeaakiabg2da9iab=j7aInaaBaaaleaacqaIZaWmaeqaaOGaeyypa0JaeGimaaJaeiykaKcaaiaaxMaacaWLjaWaaeWaaeaacqaIYaGmaiaawIcacaGLPaaaaaa@6421@

and the directionality (*genotype reduced-model score*) according to

log⁡10P(Ti|Qj,Tj,θ)P(Ti|Qj,Tj,θ:β2=β3=0)     (3)
 MathType@MTEF@5@5@+=feaafiart1ev1aaatCvAUfKttLearuWrP9MDH5MBPbIqV92AaeXatLxBI9gBaebbnrfifHhDYfgasaacH8akY=wiFfYdH8Gipec8Eeeu0xXdbba9frFj0=OqFfea0dXdd9vqai=hGuQ8kuc9pgc9s8qqaq=dirpe0xb9q8qiLsFr0=vr0=vr0dc8meaabaqaciaacaGaaeqabaqabeGadaaakeaacyGGSbaBcqGGVbWBcqGGNbWzdaWgaaWcbaGaeGymaeJaeGimaadabeaakmaalaaabaGaemiuaaLaeiikaGIaemivaq1aaSbaaSqaaiabdMgaPbqabaGccqGG8baFcqWGrbqudaWgaaWcbaGaemOAaOgabeaakiabcYcaSiabdsfaunaaBaaaleaacqWGQbGAaeqaaOGaeiilaWccciGae8hUdeNaeiykaKcabaGaemiuaaLaeiikaGIaemivaq1aaSbaaSqaaiabdMgaPbqabaGccqGG8baFcqWGrbqudaWgaaWcbaGaemOAaOgabeaakiabcYcaSiabdsfaunaaBaaaleaacqWGQbGAaeqaaOGaeiilaWIae8hUdeNaeiOoaOJae8NSdi2aaSbaaSqaaiabikdaYaqabaGccqGH9aqpcqWFYoGydaWgaaWcbaGaeG4mamdabeaakiabg2da9iabicdaWiabcMcaPaaacaWLjaGaaCzcamaabmaabaGaeG4mamdacaGLOaGaayzkaaaaaa@605B@

Moreover, if the *β*_2 _and *β*_3 _terms are weak, then it indicates that the major effect is the QTL interval and so our confidence in the *specific *regulator gene is correspondingly weak. Thus, confidence in the gene, *T*_*j*_, as the actor in the relationship is found with the *expression reduced-model score*

log⁡10P(Ti|Qj,Tj,θ)P(Ti|Qj,Tj,θ:β1=β3=0)     (4)
 MathType@MTEF@5@5@+=feaafiart1ev1aaatCvAUfKttLearuWrP9MDH5MBPbIqV92AaeXatLxBI9gBaebbnrfifHhDYfgasaacH8akY=wiFfYdH8Gipec8Eeeu0xXdbba9frFj0=OqFfea0dXdd9vqai=hGuQ8kuc9pgc9s8qqaq=dirpe0xb9q8qiLsFr0=vr0=vr0dc8meaabaqaciaacaGaaeqabaqabeGadaaakeaacyGGSbaBcqGGVbWBcqGGNbWzdaWgaaWcbaGaeGymaeJaeGimaadabeaakmaalaaabaGaemiuaaLaeiikaGIaemivaq1aaSbaaSqaaiabdMgaPbqabaGccqGG8baFcqWGrbqudaWgaaWcbaGaemOAaOgabeaakiabcYcaSiabdsfaunaaBaaaleaacqWGQbGAaeqaaOGaeiilaWccciGae8hUdeNaeiykaKcabaGaemiuaaLaeiikaGIaemivaq1aaSbaaSqaaiabdMgaPbqabaGccqGG8baFcqWGrbqudaWgaaWcbaGaemOAaOgabeaakiabcYcaSiabdsfaunaaBaaaleaacqWGQbGAaeqaaOGaeiilaWIae8hUdeNaeiOoaOJae8NSdi2aaSbaaSqaaiabigdaXaqabaGccqGH9aqpcqWFYoGydaWgaaWcbaGaeG4mamdabeaakiabg2da9iabicdaWiabcMcaPaaacaWLjaGaaCzcamaabmaabaGaeGinaqdacaGLOaGaayzkaaaaaa@605B@

And so a hypothesis of a causal regulator-target relationship requires significant values from the full model and reduced-model scores (equations 2, 3, and 4). However, interesting results can be achieved when some, but not all, scores are significant. For example, assuming that *T*_*j *_controls *T*_*i*_, there may be a functional polymorphism in gene *j'*s sequence, but no observed variation in expression level, *T*_*j*_. In that case, the expression reduced-model score will not be significant and we cannot confidently implicate gene *j *versus its nearby linked neighbors. On the other hand, suppose there is no polymorphism in gene *j*, but expression levels *T*_*i *_and *T*_*j *_are highly correlated. Then the genotype reduced-model score will not be significant, but the expression reduced-model score will be significant. In such a case, we can infer that a relationship exists between genes *i *and *j*, but cannot make any claim about the type of relationship, which could be causal in either direction or driven by a hidden actor.

### Cases 2 and 3: cis- and cis-trans-acting Regulators

Since we are interested here in fine mapping, we specifically avoid in this analysis modeling the *cis *control of target or regulator, directly or indirectly, i.e. the *cis *and *cis-trans *models. Such *cis *effects can be detrimental to fine mapping because linkage disequilibrium causes transcripts in local regions to be highly correlated. Disambiguating among different neighboring genes under *cis-trans *control can be accomplished, in principle, as implied by figure 1e, by conditioning on the genotype, but we don't pursue that further in this paper. Such a *cis-trans *local linkage problem can be framed as a kind of pleiotropy test and attempts are described elsewhere for fine mapping in such contexts [19].

### Mapping and structure inference

**Figure 3:**
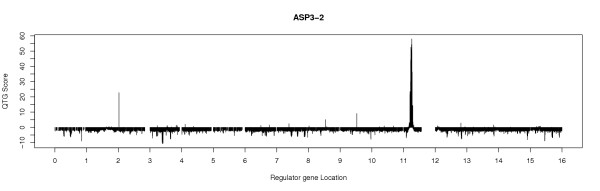
**Sample QTG linkage map**. A sample QTG linkage map on the yeast genome using the *trans*- model for the target gene ASP3-2. A separate permutation test was performed per site and the corresponding threshold was subtracted from the full model score. Thus, positive values represent locally significant results.

Our model can be used to produce a QTG map (figure 3) for each target gene. These maps are similar to conventional QTL maps, but differ in that peaks are usually narrow (unless confounded by local linkage) and there is no genome-wide LOD significance threshold. Instead, the local significance threshold at each test site is subtracted from the LOD score such that positive values are significant.

**Figure 4:**
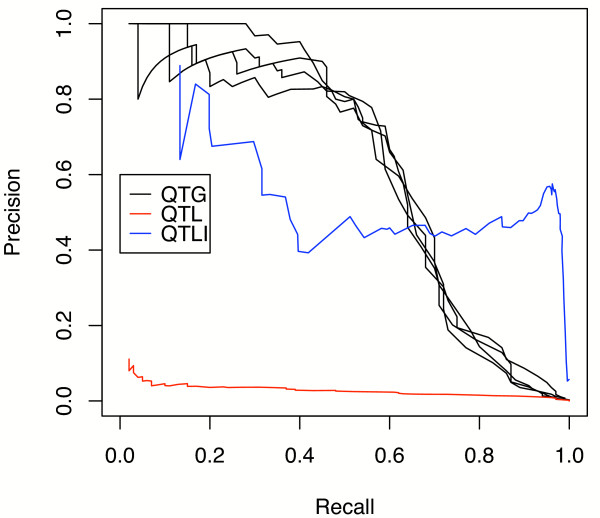
**Multiple regulators**. A plot of recall (TPTP+FN)
 MathType@MTEF@5@5@+=feaafiart1ev1aaatCvAUfKttLearuWrP9MDH5MBPbIqV92AaeXatLxBI9gBaebbnrfifHhDYfgasaacH8akY=wiFfYdH8Gipec8Eeeu0xXdbba9frFj0=OqFfea0dXdd9vqai=hGuQ8kuc9pgc9s8qqaq=dirpe0xb9q8qiLsFr0=vr0=vr0dc8meaabaqaciaacaGaaeqabaqabeGadaaakeaadaqadaqaamaalaaabaGaemivaqLaemiuaafabaGaemivaqLaemiuaaLaey4kaSIaemOrayKaemOta4eaaaGaayjkaiaawMcaaaaa@3615@ precision (TPTP+FP)
 MathType@MTEF@5@5@+=feaafiart1ev1aaatCvAUfKttLearuWrP9MDH5MBPbIqV92AaeXatLxBI9gBaebbnrfifHhDYfgasaacH8akY=wiFfYdH8Gipec8Eeeu0xXdbba9frFj0=OqFfea0dXdd9vqai=hGuQ8kuc9pgc9s8qqaq=dirpe0xb9q8qiLsFr0=vr0=vr0dc8meaabaqaciaacaGaaeqabaqabeGadaaakeaadaqadaqaamaalaaabaGaemivaqLaemiuaafabaGaemivaqLaemiuaaLaey4kaSIaemOrayKaemiuaafaaaGaayjkaiaawMcaaaaa@3619@ for varying full model scores. **QTG**: our model; a positive classification is a regulator whose score exceeds the threshold; Multiple plots for QTG are shown for *k *= 2 ... 5 as the number of additional regulators in the regulon, i.e. extra noise terms. **QTL**: the conventional QTL score where a positive is measured as with QTG; **QTLI: **an easier test using the QTL score where a true positive is called when the true regulator gene is found within the QTL interval.

**Figure 5:**
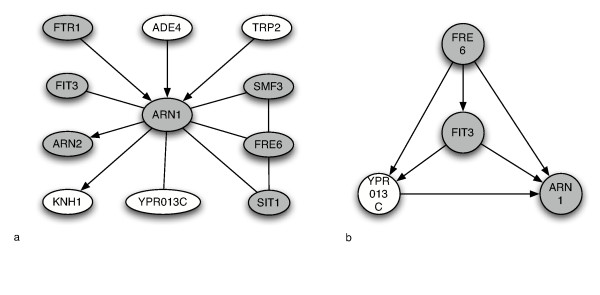
**ARN1 Regulatory module**. On the left, a QTG network built from confident pairwise relationships with respect to the seed ARN1 gene involved in iron homeostasis. Undirected edges indicate significant full model scores, but not significant genotype reduced-model scores. Directed edges identify causal relationships. Shaded nodes are known to be involved in iron homeostasis. On the right, a BN constructed from discretized gene expression alone. 2,3, and 4 bin discretizations yielded the same results.

In addition to basic mapping, the QTG model described above leads us to propose a seed-based partially directed graph of regulatory relationships built from confident Markov pairs, much like the regulatory modules studied by [14] and others. Deriving such a sub-network is computationally tractable and biologically relevant, since most biological analysis is concerned with a pathway centered around a gene of interest. Such a seed-based approach yields a network of dependent genes as nodes whose edges are directed when causal influence is significant and undirected otherwise (figure 5). (Inferring a complete network is not reasonable for any single data set where the perturbations are incomplete and the data is too sparse. In particular, with genotype/expression data sets, regardless of the number of matings, without allele-specific differential expression no characterization of a target gene can be inferred. Without polymorphisms in upstream candidate regulators, no relationships can be hypothesized.)

## Discussion and Conclusion

We proposed an improved, principled method for mapping causal loci involved in transcriptional control when analyzing data sets of whole genome genotype and expression data. Our "QTG" model is a natural application of epistatic models to gene expression, allowing for interactions among gene expression and genotype. From a genetics perspective, our model is a more complex scoring function for the identification of a single, causative gene instead of the conventional multi-gene QTL interval. And from a bioinformatics perspective, it is an improved score function for BN regulatory module reconstruction. Here we considered the simplest question of detecting relationships between regulator-target pairs, but we plan to extend this to the construction of Markov blankets.

We only presented results for the sub-model describing *trans*-acting genes, but the generality of the QTG model allows for incorporation of *cis*-acting effects for targets and regulators. Of note, when all polymorphic sites are known, but the haplotype is unknown for any sample, as is the case when crossing two fully sequenced genomes, then one may choose the full model or a sub-class of the full model as appropriate. For example, if there is no polymorphic sites in the non-coding regions of the target gene then the *cis*-acting model parameters can be dropped since the genotype is likely to be uninfor-mative.

We contrast this work with other related efforts as follows. First, other integrative work has attempted to incorporate interactions among variables. For example, Jiang and Zeng [33] describe a general method to simultaneously fit multiple effects to a common QTL by introducing multiple dependent variables and leveraging the covariance structure among them. Such an approach could theoretically be used to more confidently map pleiotropic loci. In our method, we instead introduce interactions among multiple regressors, namely the simultaneous consideration of genotype and expression of a regulator gene. Second, methods such as [20] and [21] have attempted to finely map regulators by first selecting candidate QTLs and then identifying candidate genes within these QTLs by expression correlation. Our main contribution is that we show how to do this simultaneously, and thereby consider the interacting effects that allow for fine mapping. Lastly, work by Schadt and colleagues infer causality by applying conditional independence [19] or by placing priors on causal relationships according to the numbers of QTLs and the location of the transcripts with respect to their QTLs [18]. We do not consider conditional independence or QTL count arguments, but do consider specifically the relative location of QTLs and transcripts as in [18]. The main difference is that we eliminate confounding effects from cis-QTLs and close linkage and that our score function incorporates interacting effects.

There are significant limitations to our model. With small sample sizes, many relations will go undetected. Small sample size also emphasizes linkage effects, which undermine our model. Nearby genes may appear to be regulator-target pairs due to close linkage. Relatedly, regulators that are themselves under *cis *control have strong linkage effects on neighboring genes, which makes fine mapping impossible, as was demonstrated on the Brem candidate loci. In practice, we recommend excluding candidate regulator-target pairs sharing the same chromosome and excluding regulators that are *cis*-regulated themselves.

Another major source of difficulty is missing (latent) variables. We assume that all expressed transcripts are known and correctly measured by the mi-croarray. In fact, even in yeast, as much as 5% of the proteome is believed to be unannotated small protein-coding genes [34]. Other non-coding RNA genes, including miRNAs, are believed to play a large role in transcriptional regulation. Protein expression is assumed to correspond to mRNA abundance, but overall correlation has been shown to be only 0.66 between yeast mRNA and protein products [35]. And, of course, the true dynamics of transcriptional control are rate-based. Latent variables and kinetics can be included in BN modeling [36], but at the expense of additional complexity, which requires more samples for parameter estimation. On the bright side, when variables are missing from expression data, it has been shown that predicted network structures usually roughly approximate the correct solution [37].

With the finer mapping precision of the QTG model and judicious filtering, we found some functional enrichment among regulators that had not been observed in previous reports. However, as was reported elsewhere, we confirmed that transcription factors were not among the enriched functional classes, which indicates the degree of complexity of transcriptional control even in yeast.

## Authors' contributions

DK conceived of the study, participated in the development of the methodology, and wrote the manuscript. MJ participated in the developmentof the methodology, implemented the software, and performed the tests and data analysis. Both authors read and approved the final manuscript.
